# Supplementation of lamb diets with vitamin E and rosemary extracts on meat quality parameters

**DOI:** 10.1002/jsfa.10319

**Published:** 2020-02-24

**Authors:** Leonel N Leal, José A Beltrán, Marc Bellés, José M Bello, Leo A den Hartog, Wouter H Hendriks, Javier Martín‐Tereso

**Affiliations:** ^1^ Trouw Nutrition Research and Development Amersfoort Netherlands; ^2^ Animal Nutrition group, Department of Animal Sciences Wageningen University & Research Wageningen Netherlands; ^3^ Departamento de Producción Animal y Ciencia de los Alimentos, Facultad de Veterinaria Instituto Agroalimentario de Aragón (IA2) Zaragoza Spain; ^4^ Nanta S.A. Madrid Spain

**Keywords:** vitamin E, *all*‐*rac*‐*α*‐tocopheryl acetate, rosemary extract, lipid oxidation, colour, modified‐atmosphere packaging

## Abstract

**BACKGROUND:**

Supranutritional supplementation of lamb diets with *α*‐tocopherol is an effective method to reduce lipid oxidation and colour deterioration in meat products. However, alternative antioxidant sources have been proposed to replace the supranutritional vitamin E applications.

**RESULTS:**

Indoor concentrate‐fed Rasa Aragonesa male lambs (*n* = 480) were supplemented with increasing levels of *all‐rac*‐*α*‐tocopheryl acetate (0.25, 0.5, 1.0 g kg^−1^ compound feed), rosemary extract (0.20, 0.40, or 0.80 g kg^−1^ compound feed), or rosemary extract embedded in a fat matrix (0.20, 0.40, or 0.80 g kg^−1^ compound feed) for 14 days before slaughter. The *longissimus thoracis et lumborum* muscle from three lambs per pen (18 lambs per treatment) were modified‐atmosphere packaged (70% O_2_ + 30% CO_2_) and maintained under retail conditions for 14 days. Supranutritional supplementation with antioxidants had no effect (*P* > 0.05) on average daily weight gain, feed intake, and feed efficiency. Rosemary extract supplementation (with or without fat embedment) had no effect on lipid oxidation, myoglobin forms, or colour stability parameters, regardless of the dose. All vitamin E supplementation levels significantly affected lipid oxidation, colour stability (L*, C*, and *h*), myoglobin forms, and meat discoloration parameters compared with non‐supplemented lambs.

**CONCLUSIONS:**

This study demonstrates that, unlike vitamin E, neither dose nor protection of the rosemary extract had an effect on lipid oxidation or meat colour stability of lambs during the 14 days of storage under retail conditions. © 2020 The Authors. *Journal of The Science of Food and Agriculture* published by John Wiley & Sons Ltd on behalf of Society of Chemical Industry.

## INTRODUCTION

Appearance, texture, and flavour are important quality attributes influencing the consumer's choice to purchase meat products.^1^ In lamb meat, post‐mortem biochemical changes, such as lipid oxidation, lead to off‐odours and flavour development that have a negative impact on the shelf life of these products.^2^ Therefore, the possibility to extend shelf life and subsequent display time in lamb meat is a primary objective of the meat industry.

Dietary supplementation of lamb diets with vitamin E, and more specifically *α*‐tocopherol, raises the concentrations of *α*‐tocopherol in lamb tissues,^3^, ^4^ which in turn delays lipid oxidation and improves the colour stability of the meat.^5^, ^6^, ^7^ The protective role of *α*‐tocopherol against lipid oxidation in lamb meat is well established. However, there is still a debate on the minimum dose of vitamin E in lamb diets that effectively protects the meat from lipid deterioration and values ranging from 287 and 1000 mg kg^−1^ feed have been proposed.^5^, ^7^, ^8^, ^9^, ^10^ Any recommendation on vitamin E needs to consider the wide variation of *α*‐tocopherol concentration in feedstuffs,^11^ vitamin E status of the animal at the start of the supplementation period,^12^ length of the supplementation period,^9^ differences in slaughter weight,^13^ and animal breed.^7^ In Mediterranean farming systems, lambs are typically weaned around 12–14 kg of body weight (BW), and afterwards fed *ad libitum* concentrates plus straw until they reach 22–24 kg BW.^14^ Under these conditions, Gonzalez‐Calvo *et al*.^9^ found that a concentrate supplemented with 500 mg of vitamin E per kilogram, fed for a period of 7–14 days before slaughter, was sufficient to protect lamb meat from lipid oxidation and metmyoglobin (MetMb) formation.

Alternative antioxidant sources to vitamin E from several types of plants and plant materials have been investigated to replace vitamin E. However, to effectively replace supranutritional vitamin E applications, these plant‐derived antioxidants need to be effectively absorbed, transported, and have a considerable deposition into the lamb muscle. In recent years, special attention has been given to rosemary (*Rosmarinus officinalis* L.) products and by‐products, such as leaves, essential oils, distilled leaves, and extracts from the second distillation of rosemary leaves with different solvents.^15^, ^16^, ^17^, ^18^, ^19^ Early work from Nieto *et al*.^16^ found that supplementation of pregnant ewes with 100 g of rosemary leaves kilogram feed during pregnancy and lactation, improved the shelf life of lamb meat. Interestingly, two major diterpenes present in rosemary, carnosic acid and carnosol, were later identified in lamb muscle at concentrations that could potentially lead to an antioxidant and antimicrobial effect in the meat.^20^ Supplementation of lamb diets with rosemary by‐products has yielded some conflicting results,^19^, ^21^, ^22^ probably due to a lack of product standardization^17^ or because of potential rumen degradation.^23^, ^24^

There are only a few studies comparing supranutritional doses of vitamin E with rosemary by‐products on their capacity to protect lamb meat from oxidative deterioration.^17^, ^25^ Moreover, none of the studies performed a dose titration for both antioxidant sources in order to determine the relative value of each supplementation strategy on meat quality parameters.

The primary aim of the present study is to compare increasing dosages of a standardized rosemary extract (RE) with vitamin E (*all‐rac*‐*α*‐tocopheryl acetate) in their ability to improve meat colour and stability parameters in lamb meat stored for 14 days under common retail conditions. Second, as a means to mitigate the degradation of the RE by rumen microorganisms, a standardized plant extract embedded in a rumen‐inert fat will be also tested.

## MATERIALS AND METHODS

The animal care and slaughter procedures used met the guidelines of Council Directive 86/609/EEC (European Communities, 1986) on the protection of animals used for experimental and other scientific purposes.

### Animals and diets

A total of 480 Rasa Aragonesa male lambs were used in this study (BW = 21.8 ± 1.39 kg). Every 3 weeks, lambs were purchased from local dealers and incorporated in the experiment in three separated batches of 160 animals each. Upon arrival at the commercial farm Franco y Navarro (Zaragoza, Spain), lambs were allocated based on BW to 20 concrete pens (20 m^2^) bedded with straw. For 14 days before slaughter, the lambs had free access to the experimental compound feed (presented as 3.5 mm diameter pellet; Table 1), barley straw, and water via separated troughs. Per batch, two pens were randomly assigned to one of 10 treatments consisting of a basal compound feed (control), supplemented with increasing levels of *all‐rac*‐*α*‐tocopheryl acetate (0.25, 0.50, or 1.0 g kg^−1^ compound feed), RE (Nutrox OSP; Nutrafur S.A., Murcia, Spain; 0.20, 0.40, or 0.80 g kg^−1^ compound feed), or the same RE embedded in a fat matrix (FRE; 0.20, 0.40, or 0.80 g kg^−1^ compound feed).

**Table 1:** Composition of the basal diet and experimental premix

Ingredients (g kg^−1^)			
Barley	295	Limestone	25
Soya bean meal (480 g kg^−1^ of crude protein)	240	Experimental premix	10
Wheat	200	Sodium bicarbonate	7
Maize	200	Sodium chloride	5
Soya oil	15	Standard mineral and vitamin premix^a^	3

		SE			RE			FRE		
Experimental premix	Control	250	500	1000	200	400	800	200	400	800
Ingredients, g kg^−1^										
Wheat bran	560	560	560	560	560	560	560	560	560	560
Hydropalm^b^	140	140	140	140	140	140	140	105	70	
Silica^c^	300	250	200	100	280	260	220	255	210	120
Rosemary^d^					20	40	80			
Fat‐embedded rosemary^e^								80	160	320
*all‐rac*‐*α*‐Tocopheryl acetate^f^		50	100	200						

a Mineral and vitamins provided: calcium 0.24 g, sodium 0.47 g, sulfur 0.23 g, manganese 30 mg, zinc 50 mg, copper 5 mg, iodine 0.5 mg, cobalt 0.5 mg, selenium 0.15 mg, iron 50 mg, vitamin A 8000 IU, vitamin D_3_ 1600 IU, *all‐rac*‐*α*‐tocopheryl acetate 25 mg. SE: *all‐rac*‐*α*‐tocopheryl acetate. RE: rosemary extract. FRE: fat‐embedded rosemary extract.

b Hydropalm: hydrogenated palm fatty acids (Norel, Spain).

c Silica: silica H_2_O (Trouw Nutrition, Netherlands).

d Rosemary: standardized rosemary extract (Nutrafur S.A., Spain).

e Fat‐embedded rosemary: standardized rosemary extract (inclusion of 25%) mixed with melting (70 ± 2 °C) hydropalm (43.75%) and silica (31.25%).

f *all‐rac‐α*‐Tocopheryl acetate: contains 50 g *all‐rac*‐*α*‐tocopherol per 100 g of product (Trouw Nutrition, Netherlands).

The different antioxidant sources were included in the compound feed via a treatment‐specific experimental premix (Table 1). To produce the FRE, hydrogenated palm fatty acids (Norel, Madrid, Spain) were melted at 70 ± 2 °C. Afterwards, the RE (Nutrox OSP; Nutrafur S.A.) was added to the melted hydrogenated palm fatty acids and stirred for 10 min at 70 ± 2 °C, until an homogeneous mixture was obtained. The mixture of hydrogenated palm fatty acids and RE was then added to silica (Trouw Nutrition, Amersfoort, Netherlands), stirred for 20 min and cooled at room temperature. The final product (free‐flowing powder) was composed of 43.75% hydrogenated palm fatty acids, 31.25% silica, and 25.0% RE.

After 14 days of exposure to the treatment diets and following an overnight period without feed (but with free access to water), lambs from all treatments were mixed in a single group, transported in the same lorry, and slaughtered at the local abattoir (Mercazaragoza S.A., Zaragoza, Spain) within 2 h after leaving the farm.

### Meat processing and packaging

Three lambs per pen (in total, 18 lambs per treatment) were randomly selected at the start of the experimental period for meat analysis. The carcasses were chilled for 24 h at 2 °C before being split longitudinally into two halves. The *longissimus thoracis et lumborum* (LTL) muscle from the right half was dissected, with subcutaneous fat removed, placed in bags (one muscle per bag), and transported in sealed plastic containers in darkness at 4 ± 1 °C to the Meat Quality Laboratory of the Veterinary Faculty of Zaragoza (Zaragoza, Spain).

Within 2 h after arrival, the LTL muscles were sectioned into approximately 1.5 cm thick portions, which were placed in polystyrene trays B5‐37 (Aerpack), packed under modified‐atmosphere packaging with 70% oxygen (O_2_) + 30% carbon dioxide (CO_2_) (Ulma Smart 500; Ulma Packaging, Guipúzcoa, Spain), and sealed with a polyethylene and polyamine laminate film (30 μm thickness, water vapour transmission rate at 23 °C of <7 g m^−2^ per 24 h at 85% relative humidity (RH), an O_2_ transmission rate at 23 °C of <15 cm^3^ m^−2^ at 0% RH, and a CO_2_ transmission rate at 23 °C of <75 cm^3^ m^−2^ per 24 h at 0% RH; Linpac Packaging S.L., Spain). The trays were stored at 4 ± 1 °C for 1, 7, 9, 12, or 14 days in a Zafrio display cabinet (Zafrio, Zaragoza, Spain), simulating retail conditions with a daily exposure to 14 h of light at 1200 lx.

### Physical and chemical analysis

Colour was measured using a reflectance spectrophotometer (CM‐2002; Minolta, Osaka, Japan) directly on the meat surface, after 2 h exposure to air. Each value was the mean of 10 determinations per sample. The parameters recorded according to the CIELAB system,^26^ were lightness L*, redness a*, and yellowness b*. Values of chroma C* and hue angle *h* were calculated as C* = (a* ^2^ + b* ^2^)^0.5^, and *h* = tan^−1^(b*/a*), expressed in degrees.

Lipid oxidation was expressed as thiobarbituric acid reactive substances (TBARS) and expressed as milligrams of malondialdehyde (MDA) per kilogram of meat, as described by Pfalzgraf *et al*.^27^ Briefly, 10 g of meat sample was homogenized with 10% trichloroacetic acid using an Ultra‐Turrax T25 (Janke & Kunkel, Staufen, Germany). After centrifugation at 1500×*g* for 30 min (at 10 °C) the supernatant was filtered (Filterlab, Barcelona, Spain), 2 mL of the filtrate was mixed with 2 mL of thiobarbituric acid (20 mol L^−1^), homogenized, and incubated for 20 min (in boiling water). Absorbance was measured at 532 nm, and TBARS values were calculated from a standard curve of MDA obtained by the hydrolysis of 1,1,3,3‐tetramethoxypropane (Sigma‐Aldrich, Madrid, Spain). Samples were analysed in duplicate, and the results are expressed as milligrams of MDA per kilogram of meat.

Myoglobin forms (deoxymyoglobin, oxymyoglobin (OxyMb), and MetMb) were calculated from the reflectance curve described by Krzywicki^28^ using a wavelength of 690 nm. The reflectance at 473, 525, and 572 nm was obtained by linear interpolation, since the reflectance spectrophotometer only measures the reflectance between 400 and 740 nm at intervals of 10 nm.

The rate of meat discoloration was accessed by the parameter *A*_580_ − *A*_630_, according to the method proposed by van den Oord and Wesdorp^29^ Oxygen saturation of myoglobin on meat surface *I*_SO2_ was measured following the technique described by Tsuruga *et al*.^30^

### Polyphenol analysis in feed

Feed samples were homogenized using a blender and sieved with a no. 18 mesh (corresponding to 1 mm) to remove large particles before extraction. The phenolic compounds were extracted using methanol:water (80:20, v:v), sonicated for 5 min, and centrifuged. This procedure was repeated twice with the feed residue. The supernatants were combined and the methanol evaporated. For better chromatography resolution, the extracts were cleaned using solid‐phase extraction with Oasis MAX 96‐well plates (30 mg, 30 μm) from Waters (Water corporation, Massachusetts, USA), according to Vallverdú‐Queralt *et al*.^31^

The identification and quantification of the major phenolic compounds was performed using ultrahigh performance liquid chromatography (UHPLC; Waters Acquity Ultra Performance LC) coupled to an API 3000 triple quadrupole mass spectrometer (ABSciex) equipped with a Turbo ion spray source operating in negative mode. Separation was carried out using a BEH C18 (2.1 mm × 50 mm, 1.7 μm; Acquity UHPLC) maintained at 30 °C. Gradient elution was performed with acetonitrile with 0.1% of formic acid (v/v) and water with 0.1% of formic acid (v/v) using an increasing linear gradient flow of acetonitrile with 0.1% of formic acid (v/v) under the following conditions: 0 min, 20%; 0.5 min, 20%; 1.5 min, 30%; 2 min, 30%; 2.5 min, 50%; 3 min, 100%; 3.5 min, 100%; 3.7 min, 20%; and 4.5 min, 20%. The flow rate was 0.4 mL min^−1^ and the injection volume was 10 μL. The identification and quantification of each compound were carried out using a mass spectrometer in multiple reaction monitoring mode. The quantification of the phenolic compounds was performed using calibration curves with analytical standards with the internal standard method. The internal standard was ethyl gallate (400 ng g^−1^) (Extrasynthese, Genay, France) and the results are expressed as milligrams per kilogram of feed.^32^

### Statistical analysis

All statistical analyses were performed using SAS Studio (SAS Institute, Inc., Cary, NC, USA). Individual lamb data were summarized per pen, which was considered as the experimental unit for all of the parameters studied.

The model included the fixed effects of antioxidant supplementation, display time, and the interaction between antioxidant supplementation and display time. The effect of batch (three rounds of 180 lambs each) was initially included in the statistical model as a fixed effect, but it was finally excluded as batch never reached statistical significance (*P* > 0.05). Therefore, the model was

$$Y_{\mathrm{ijk}}=\mu +{\mathrm{AS}}_{i}+D_{j}+\left({\mathrm{AS}}_{i}\times D_{j}\right)+e_{\mathrm{ijk}}$$

where *Y*_*ijk*_ is the dependent variable, *μ* is the population average, AS_*i*_ is the fixed effect of antioxidant supplementation, *D*_*j*_ is the fixed effect of display time, AS_*i*_ × *D*_*j*_ is the interaction between AS_*i*_ and *D*_*j*_, and *e*_*ijk*_ is the random error. Differences were declared significant when *P* < 0.05. Tukey's post hoc test was used to assess differences between mean values when *P* < 0.05.

## RESULTS AND DISCUSSION

### Animal performance

Data on BW, average daily weight gain, feed intake, and feed efficiency are presented in Table 2. There were no effects of either antioxidant source (vitamin E and RE) or dosage in the assessed performance parameters. These results align with previous findings in which dietary vitamin E inclusion levels up to 2.0 g kg^−1^ feed had no effect on lamb performance.^3^, ^5^ Moreover, supplementation of lamb diets with rosemary diterpenes^17^ (0.6 g kg^−1^ diet) or rosemary essential oils^19^ (0.3 or 0.6 mL day^−1^) were also found to have no effect on growth, feed intake, or efficiency.

**Table 2:** Productive performance of the lambs fed increasing level of *all‐rac*‐*α*‐tocopheryl acetate (SE), rosemary extract (RE), or fat‐embedded rosemary extract (FRE)

		SE (mg kg^−1^ feed)			RE (mg kg^−1^ feed)			FRE (mg kg^−1^ feed)			SEM	*P* value
Item	Control	250	500	1000	200	400	800	200	400	800		
IBW (kg)	21.9	21.8	21.8	21.9	21.9	21.9	21.8	21.8	21.8	21.9	0.04	0.746
FBW (kg)	26.4	26.2	26.5	26.4	26.2	26.7	26.5	26.4	26.3	26.5	0.29	0.848
FEED, (kg day^−1^)	0.992	0.996	1.005	1.015	1.006	1.039	1.032	1.025	1.029	1.027	0.0362	0.880
ADG (kg day^−1^)	0.329	0.315	0.331	0.324	0.315	0.351	0.331	0.323	0.319	0.331	0.0205	0.832
FCR (kg kg^−1^)	3.11	3.25	3.07	3.17	3.20	3.07	3.21	3.15	3.40	3.21	0.191	0.824

ADG: average daily gain; FBW: final body weight; FCR: feed conversion ratio; FEED: compound feed intake; IBW: initial body weight; SEM: standard error of the mean.

### Polyphenol content in feeds and fat embedment

The identification and quantification of the major polyphenols present in the experimental diets are shown in Table 3. Overall, we found high concentrations of chlorogenic acid, ferulic acid, and ferulic acid‐*O*‐hexoside, which are commonly found in cereals,^33^ which accounted for >42% of the total polyphenol content in the experimental diets. Diets containing REs (either protected or unprotected), provided more carnosol, carnosic, and rosmarinic acid, major polyphenols present in REs,^34^ than the control diet. According to the information provided by the supplier, the RE contained approximately 20% diterpenes, in a ratio of 2:1 between carnosic acid and carnosol. However, RE supplementation (RE and FRE) resulted in higher concentrations of carnosol than carnosic acid in the experimental feeds. According to Jordán *et al*.,^20^ carnosol is a major component produced in the carnosic acid oxidation pathway, which could explain the high carnosol concentrations in the rosemary‐supplemented diets and the relative low amounts of carnosic acid. On the other hand, the lower amounts of carnosol and carnosic acid in FRE diets compared with RE diets could be associated with the extra heat treatment^35^ applied to the FRE diets during the incorporation of the RE into the melted fat (70 ± 2 °C).

**Table 3:** Quantification of individual bioactive compounds (mg kg^−1^) of the different feeds

		SE (mg kg^−1^ feed)			RE (mg kg^−1^ feed)			FRE (mg kg^−1^ feed)		
Item	Control	250	500	1000	200	400	800	200	400	800
CA	261	357	223	255	264	314	266	235	185	265
CrA	13	13	13	13	23	19	24	25	19	27
Cn	11	8	6	10	1366	1383	2149	862	553	551
ChA	1060	1084	801	837	782	634	755	777	498	742
DA	55	7	43	19	18	15	22	11	12	28
FA	926	845	810	716	753	675	872	785	594	851
FAH	1593	1423	1401	1264	1375	858	1677	1649	1035	1761
N	60	67	67	68	62	64	63	69	55	60
NG	373	392	367	355	362	338	379	379	292	363
pCA	596	491	489	429	436	444	522	480	356	528
PA	166	228	140	154	156	166	171	159	107	158
Q	55	58	57	56	57	58	62	59	58	56
RsA	31	29	30	31	40	71	128	35	50	40
R	118	73	101	73	66	92	79	76	79	83
Total	5318	5075	4548	4280	5760	5131	7169	5601	3893	5513

CA: caffeic acid; ChA: chlorogenic acid; Cn: carnosol; CrA: carnosic acid; DA: dicaffeoylquinic acid; FA: ferulic acid; FAH: ferulic acid‐*O*‐hexoside; FRE: fat‐embedded rosemary extract; N: narigenin; NG: naringenin glucoside; PA: protocatechuic acid; pCA: *p‐*cumaric acid; Q: quercetin; R: rutin; RE: rosemary extract; RsA: rosmarinic acid; SE: *all‐rac*‐*α*‐tocopheryl acetate.

### Lipid oxidation

Mean TBARS values for each dietary treatment during the 14 days of display are presented in Table 4. Lipid oxidation significantly increased with storage time in all groups (*P* < 0.05). Moreover, an interaction between storage time and dietary treatment was found (*P* < 0.001) for TBARS values. From day 7 onwards, LTL muscle from vitamin‐E‐supplemented lambs (irrespective of dose) presented significantly lower TBARS values than the control and RE‑ and FRE‐supplemented lambs. Vitamin‐E‐supplemented lambs presented TBARS values ranging from 0.16 to 0.31 mg MDA kg^−1^ meat on day 7, whereas the remaining treatments registered TBARS values between 1.63 and 1.99 mg MDA kg^−1^ meat. With increasing storage time, the differences between the vitamin‐E‐supplemented lambs and the control, RE, and FRE lambs became wider. On day 14 of storage, LTL muscle from control and the rosemary‐supplemented lambs (RE and FRE) presented TBARS values ranging from 3.25 to 4.00 mg MDA kg^−1^ meat, whereas vitamin‐E‐supplemented LTL muscles showed TBARS values from 0.39 to 0.69 mg MDA kg^−1^ meat. In concentrate‐fed lambs, slaughtered at low BWs (lower than 30 kg live weight), a threshold of 1.0 mg MDA kg^−1^ meat has been proposed to be avoided for the development of off flavours in lamb meat.^36^ After 7 days of storage, higher MDA values than 1.0 mg kg^−1^ meat were found in the LTL muscle from the control, FRE, and RE lambs. Interestingly, vitamin E supplementation at 0.25, 0.50 or 1.0 g kg^−1^ feed led to lower TBARS values (0.69, 0.56, and 0.39 mg MDA kg^−1^ meat) than the proposed threshold after 14 days of storage. In a similar study, Leal *et al*.^6^ found that a supplementation level of 0.16 g kg^−1^ feed with vitamin E (*all‐rac*‐*α*‐tocopheryl acetate) for 14 days before slaughter was sufficient to maintain TBARS values below the threshold of 1.0 mg MDA kg^−1^ meat. Dietary supplementation of lamb diets with vitamin E is a consistent strategy to increase *α*‐tocopherol content in lamb tissues like muscle,^3^ and to improve the resistance of lamb meat to oxidative deterioration.^5^, ^6^, ^37^

**Table 4:** Effect of dietary supplementation^a^ with *all‐rac*‐*α*‐tocopheryl acetate (SE), rosemary extract (RE), and fat‐embedded rosemary extract (FRE) on thiobarbituric acid reactive substances in raw lamb meat stored in modified‐atmosphere packaging (70% O_2_:30% CO_2_) kept for 1, 7, 9, 12, and 14 days under retail conditions

Item	Days of display					SEM	*P*‐value display
	1	7	9	12	14		
Control	0.13^c,x^	1.90^b,y^	2.66^b,yz^	3.44^b,z^	3.80^b,z^	0.25	<0.001
SE 250	0.09^ab,x^	0.31^a,xy^	0.32^a,xy^	0.41^a,y^	0.69^a,z^	0.06	<0.001
SE 500	0.08^a,x^	0.21^a,y^	0.26^a,y^	0.30^a,y^	0.56^a,z^	0.03	0.001
SE 1000	0.09^ab,x^	0.16^a,xy^	0.18^a,xy^	0.33^a,yz^	0.39^a,z^	0.04	0.017
RE 200	0.11^abc,w^	1.99^b,x^	2.85^b,xy^	3.53^b,yz^	4.00^b,z^	0.23	<0.001
RE 400	0.11^abc,w^	1.91^b,x^	2.75^b,xy^	3.61^b,yz^	3.85^b,z^	0.21	<0.001
RE 800	0.11^abc,w^	1.86^b,x^	2.55^b,xy^	3.64^b,yz^	3.80^b,z^	0.24	<0.001
FRE 200	0.11^abc,x^	1.67^b,y^	2.46^b,yz^	2.80^b,yz^	3.25^b,z^	0.30	<0.001
FRE 400	0.11^abc,w^	1.63^b,x^	2.29^b,xy^	3.27^b,yz^	3.51^b,z^	0.23	<0.001
FRE 800	0.11^abc,x^	1.74^b,y^	2.50^b,yz^	3.25^b,z^	3.53^b,z^	0.23	<0.001
*P*‐value treatment	0.016	<0.001	<0.001	<0.001	<0.001		

a SE 250, SE 500, and SE 1000: 250, 500, and 1000 mg *all‐rac*‐*α*‐tocopheryl acetate kg^−1^ feed; RE 200, RE 400, and RE 800: 200, 400, and 800 mg rosemary extract kg^−1^ feed; FRE 200, FRE 400, and FRE 800: 200, 400, and 800 mg fat‐embedded rosemary extract kg^−1^ feed.

^a,b,c^Values within a column with different superscript are significantly different (*P* < 0.05). ^w,x,y,z^Values within a row with different superscript are significantly different (*P* < 0.05). SEM: standard error of the mean.

Unlike vitamin E, the effects of REs or essential oils on meat oxidative stability are not consistent. Shelf‐life studies in lambs have demonstrated an improvement in lipid stability of meat when rosemary by‐products were included in the diet of lactating ewes^16^ or directly into lamb diets.^15^, ^38^ In the current study, supplementation of lamb diets with REs (RE and FRE) failed to affect the oxidative deterioration of meat. Accordingly, Smeti *et al*.^18^ and Aouadi *et al*.^21^ found that supplementing lambs with rosemary essential oils did not exert any effect on lipid peroxidation in meat. Overall, the mechanisms by which antioxidant compounds present in plant extracts can affect the oxidative deterioration of lamb meat are still unclear.^39^ Interestingly, several studies found a higher concentration of tocopherol in lamb muscle following dietary supplementation with a plant extracts or by‐product.^17^, ^40^, ^41^ This is of special relevance, because even a slight increase in *α*‐tocopherol concentration in muscle (from 0.6 to 0.9 mg kg^−1^ meat) has been shown to substantially decrease lipid oxidation at 5 and 7 days of display.^9^

### Colour stability

Results of the colour measurements are shown in Table 5. Overall, storage time significantly affected the evolution of all meat colour parameters. Moreover, interactions between the dietary treatment and storage time were registered for L* (lightness), C* (chroma), and *h* (hue angle) values (*P* < 0.001).

**Table 5:** Effect of dietary supplementation^a^ with *all‐rac*‐*α*‐tocopheryl acetate (SE), rosemary extract (RE), and fat‐embedded rosemary extract (FRE) on colour parameters (L*, C*, *h*) in raw lamb meat stored in modified‐atmosphere packaging (70% O_2_:30% CO_2_) kept for 1, 7, 9, 12, and 14 days under retail conditions

	Days of display					SEM	*P*‐value display
Item	1	7	9	12	14		
Lightness L*							
Control	40.94^x^	39.19^x^	40.17^ab,x^	45.26^b,y^	47.67^b,z^	0.269	<0.001
SE 250	40.22^yz^	36.74^w^	37.98^ab,wx^	38.78^a,xy^	40.78^a,z^	0.173	<0.001
SE 500	40.32^y^	37.85^x^	38.04^ab,x^	39.21^a,xy^	40.55^a,y^	0.256	<0.001
SE 1000	39.52^yz^	37.31^x^	37.68^a,xy^	38.71^a,xyz^	40.67^a,z^	0.221	<0.001
RE 200	40.11^x^	38.45^x^	40.00^ab,x^	44.82^b,y^	47.33^b,y^	0.359	<0.001
RE 400	39.41^x^	38.38^x^	40.24^ab,x^	45.12^b,y^	48.73^b,z^	0.406	<0.001
RE 800	39.17^wx^	38.15^w^	40.67^b,x^	44.92^b,y^	48.42^b,z^	0.252	<0.001
FRE 200	39.95^x^	38.39^x^	40.09^ab,x^	43.39^b,y^	46.49^b,z^	0.324	<0.001
FRE 400	40.85^x^	38.58^x^	39.93^ab,x^	44.19^b,y^	47.09^b,z^	0.389	<0.001
FRE 800	40.98^x^	37.85^w^	40.66^b,wx^	44.98^b,y^	48.71^b,z^	0.356	<0.001
*P*‐value treatment	0.530	0.146	0.001	<0.001	<0.001		
Chroma C*							
Control	13.39^x^	15.49^ab,y^	13.54^a,x^	13.15^a,x^	13.55^a,x^	0.171	<0.001
SE 250	14.84^x^	16.92^b,yz^	17.12^d,z^	16.18^c,yz^	15.94^b,y^	0.122	<0.001
SE 500	13.94	16.10^ab^	15.65^bcd^	14.95^b^	15.38^b^	0.156	0.059
SE 1000	13.85^x^	16.12^ab,y^	16.03^cd,y^	15.73^bc,y^	16.08^b,y^	0.131	0.002
RE 200	13.93	14.81^a^	13.37^a^	12.95^a^	13.77^a^	0.179	0.189
RE 400	13.97^xy^	14.66^a,y^	13.75^a,xy^	12.98^a,x^	13.74^a,xy^	0.171	0.040
RE 800	13.20^x^	15.42^a,y^	13.99^ab,x^	12.87^a,x^	13.55^a,x^	0.153	<0.001
FRE 200	14.84	14.89^ab^	14.29^abc^	13.60^a^	13.96^a^	0.162	0.245
FRE 400	13.62^xy^	15.14^a,z^	14.32^abc,yz^	12.55^a,x^	13.58^a,xy^	0.154	0.006
FRE 800	14.17^xy^	15.36^a,y^	13.86^ab,x^	12.91^a,x^	13.56^a,x^	0.156	<0.001
*P*‐value treatment	0.077	0.003	<0.001	<0.001	<0.001		
**Hue angle *h***							
Control	51.41^x^	50.64^abc,x^	58.85^b,x^	74.04^b,y^	77.15^b,y^	1.120	<0.001
SE 250	50.15^z^	44.36^a,x^	46.16^a,xy^	46.69^a,y^	48.96^a,z^	0.238	<0.001
SE 500	51.15^z^	45.78^abc,x^	45.72^a,x^	47.37^a,xy^	48.71^a,yz^	0.339	<0.001
SE 1000	50.68^z^	44.60^ab,x^	46.25^a,xy^	47.10^a,xy^	48.17^a,yz^	0.288	<0.001
RE 200	50.24^x^	52.05^c,xy^	61.13^b,y^	73.90^b,z^	76.95^b,z^	1.185	<0.001
RE 400	50.38^x^	51.54^bc,x^	61.73^b,y^	76.42^b,z^	79.26^b,z^	1.145	<0.001
RE 800	50.74^x^	48.40^abc,x^	60.00^b,y^	73.98^b,z^	79.29^b,z^	0.936	<0.001
FRE 200	50.52^x^	50.74^abc,x^	57.74^b,x^	68.23^b,y^	73.35^b,y^	1.151	<0.001
FRE 400	52.30^x^	50.35^abc,x^	55.32^ab,x^	70.54^b,y^	76.03^b,y^	1.156	<0.001
FRE 800	51.08^xy^	49.94^abc,x^	58.96^b,y^	73.93^b,z^	78.78^b,z^	1.040	<0.001
*P*‐value treatment	0.883	<0.001	<0.001	<0.001	<0.001		

a SE 250, SE 500, and SE 1000: 250, 500, and 1000 mg *all‐rac*‐*α*‐tocopheryl acetate kg^−1^ feed; RE 200, RE 400, and RE 800: 200, 400, and 800 mg rosemary extract kg^−1^ feed; FRE 200, FRE 400, and FRE 800: 200, 400, and 800 mg fat embedded rosemary extract kg^−1^ feed.

^a,b,c^ Values within a column with different superscript are significantly different (*P* < 0.05). ^w,x,y,z^ Values within a row with different superscript are significantly different (*P* < 0.05). SEM: standard error of the mean.

After 12 days of storage, a clear reduction in L* values was found in the meat of vitamin‐E‐supplemented lambs (regardless of dose). When compared with the control, LTL muscle from lambs supplemented with RE or FRE presented no significant improvements in L* values. It has been previously proposed^42^ that human evaluators are not able to appreciate individual L*, a*, and b* coordinates. Therefore, hue angle *h* provides a better estimation of meat browning than individual coordinates. Like previously reported for individual L* coordinates, vitamin E supplementation significantly affected *h* development in lamb meat when compared with the control‑, RE‑, and FRE‐fed lambs. The effects on *h* value are clear after 9 days of display. No effect was found of RE supplementation in *h* values compared with the control. Chroma C* in meat, like with the L* values, was only affected by the dietary supplementations after 12 days of storage. Independently from dosage, vitamin E supplementation led to a significant increase in C* values compared with the meat of control‑, RE‑, and FRE‐fed lambs.

Overall, the positive effect of synthetic^6^, ^43^ and natural^6^ vitamin E supplementation on meat colour parameters has already been described in lambs. Supplementation of lamb diets with vitamin E levels above 0.5 g kg^−1^ feed has been associated with significant improvements in L*,^39^ *C*,* 17 and *h* values^6^ in meat, when compared with non‐supplemented lambs. However, our findings conflict with earlier studies that reported a positive effect on L*,^17^, ^38^ C*,^38^ and *h* values^38^, ^44^ in lamb meat, following a dietary supplementation with REs or their diterpenes. Like with vitamin E, for the phenolic compounds to exhibit any direct antioxidant activity post‐mortem they need to be absorbed along the gastrointestinal tract, transported in the blood, and accumulated in the target tissue (such as the muscle).^45^ Therefore, factors that can affect the concentration of phenolic compounds in muscle, such as dosage and length of the supplementation period, may explain the results in meat colour and oxidative stability of the RE‑ and FRE‐supplemented lambs. In the current study, lambs were supplemented for 14 days with a diet containing increasing levels (0.2, 0.4 or 0.8 g kg^−1^) of an RE (RE or FRE) that contained approximately 20% diterpenes (according to manufacturer's specifications). In contrast, studies that reported positive effects of supplementing lamb diets with REs (or diterpenes) on meat colour stability targeted longer supplementation periods,^15^, ^16^ higher dosages,^44^, ^46^ and/or were performed using extracts with higher purity.^17^

### Myoglobin oxidation and meat discoloration

The results of meat myoglobin (MetMb and OxyMb) oxidation are presented in Table 6. Overall, myoglobin oxidation outcomes are consistent with the colorimetric parameters previously discussed. Display time significantly affected all the myoglobin forms analysed (*P* < 0.01). Moreover, an interaction between antioxidant supplementation and storage time was found for MetMb and OxyMb (*P* < 0.001). Supplementation of lamb diets with vitamin E (0.25, 0.50 or 1.0 g kg^−1^ feed) was found to consistently affect MetMb and OxyMb oxidation after 12 days of storage. However, RE presentation form (RE or FRE) or dosage (0.20, 0.40 or 0.8 g kg^−1^ feed) had no effect on MetMb and OxyMb percentages in the LTL muscle when compared with the control. The effects of vitamin E supplementation on MetMb development are consistent with previous work in lambs,^5^, ^6^ where a clear inhibitory effect of vitamin E supplementation on MetMb formation in lamb meat was reported, especially during longer display periods. Moreover, in line with the current study, Yagoubi *et al*.^41^ found no effect of supplementing lamb diets with rosemary leaf residues on MetMb percentages in meat displayed for 9 days. The proportion of OxyMb increased significantly in all groups (*P* < 0.01) until 7 days of storage and decreased thereafter until day 14 of storage. Apart from display time, supplementation of lamb diets with vitamin E (0.25, 0.50 or 1.0 g kg^−1^ feed) significantly reduced OxyMb oxidation when compared with the other treatments after 12 days of display. These findings contrast with previous work that found no effect of vitamin E supplementation on OxyMb oxidation in lambs.^5^, ^6^ Interestingly, supplementation of lamb diets with REs (RE and FRE) had no effect on OxyMb percentages when compared with the control group. However, Yagoubi *et al*.^41^ found a clear reduction in OxyMb oxidation in the LTL muscle of Barbarine lambs supplemented with rosemary distillation residues for 77 days before slaughter.

**Table 6:** Effect of dietary supplementation^a^ with *all‐rac*‐*α*‐tocopheryl acetate (SE), rosemary extract (RE), and fat‐embedded rosemary extract (FRE) on myoglobin forms (OxyMb and MetMb) in raw lamb meat stored in modified‐atmosphere packaging (70% O_2_:30% CO_2_) kept for 1, 7, 9, 12, and 14 days under retail conditions

	Days of display					SEM	*P*‐value display
Item	1	7	9	12	14		
**OxyMb**							
Control	28.24^y^	41.68^z^	37.04^yz^	16.41^a,x^	11.85^a,x^	2.90	<0.001
SE 250	27.02^x^	42.07^z^	38.97^yz^	38.10^bc,y^	37.03^b,y^	0.86	<0.001
SE 500	22.34^x^	42.98^z^	40.59^yz^	38.60^c,yz^	36.14^b,y^	1.15	<0.001
SE 1000	25.53^x^	41.57^z^	38.97^yz^	37.90^bc,y^	37.29^b,y^	0.91	<0.001
RE 200	26.39^xy^	40.16^z^	33.22^yz^	20.03^a,wx^	12.43^a,w^	2.73	<0.001
RE 400	26.36^y^	40.98^z^	31.47^y^	15.39^a,x^	11.05^a,x^	2.18	<0.001
RE 800	23.14^z^	41.73^z^	34.46^y^	20.00^a,xy^	12.39^a,x^	2.32	<0.001
FRE 200	28.83^xy^	39.67^z^	36.16^yz^	20.53^a,wx^	16.23^a,w^	2.40	<0.001
FRE 400	28.01^xy^	39.86^z^	33.88^yz^	22.63^a,wx^	17.04^a,w^	3.00	0.005
FRE 800	24.52^x^	40.67^z^	34.99^yz^	25.93^a,wx^	13.72^a,w^	2.30	<0.001
*P*‐value treatment	0.928	0.738	0.593	<0.001	<0.001		
**MetMb**							
Control	22.73^w^	33.95^x^	46.55^b,y^	61.06^b,z^	63.56^b,z^	2.50	<0.001
SE 250	22.77^w^	29.23^x^	30.72^a,xy^	32.97^a,y^	35.98^a,z^	0.69	<0.001
SE 500	24.80^w^	28.11^wx^	30.55^a,xy^	32.74^a,yz^	36.10^a,z^	0.82	<0.001
SE 1000	23.67^w^	28.96^x^	30.55^a,xy^	32.38^a,y^	34.35^a,z^	0.62	<0.001
RE 200	23.37^x^	36.62^y^	48.54^ab,y^	60.96^b,z^	64.08^b,z^	2.78	<0.001
RE 400	24.07^w^	35.93^x^	49.51^ab,y^	64.61^b,z^	65.93^b,z^	2.26	<0.001
RE 800	21.58^w^	31.87^x^	47.19^b,y^	60.86^b,z^	65.17^b,z^	2.01	<0.001
FRE 200	22.87^x^	36.09^x^	43.34^b,y^	55.94^b,z^	60.89^b,z^	2.70	<0.001
FRE 400	24.57^x^	33.39^xy^	40.57^b,y^	57.06^b,z^	63.53^b,z^	2.64	<0.001
FRE 800	22.47^w^	34.21^x^	45.62^b,y^	60.23^b,z^	65.69^b,z^	2.02	<0.001
*P*‐value treatment	0.089	0.270	<0.001	<0.001	<0.001		

a SE 250, SE 500 and SE 1000 = 250, 500 and 1000 mg *all‐rac*‐*α*‐tocopheryl acetate kg^−1^ feed; RE 200, RE 400 and RE 800 = 200, 400 and 800 mg rosemary extract kg^−1^ feed; FRE 200, FRE 400 and FRE 800 = 200, 400 and 800 mg fat embedded rosemary extract kg^−1^ feed.

^a,b,c^ Values within a column with different superscript are significantly different (*P* < 0.05). ^w,x,y,z^ Values within a row with different superscript are significantly different (*P* < 0.05). SEM: standard error of the mean.

Meat discoloration, as assessed by the decrease in the *A*_580_ − *A*_630_ parameter, and O_2_ saturation on the meat surface *I*_SO2_ in LTL muscle are presented in Table 7. Meat discoloration and O_2_ saturation were significantly affected by storage time in all the groups (*P* < 0.001). Besides that, an interaction between antioxidant supplementation and storage time was found for the *A*_580_ − *A*_630_ and *I*_SO2_ parameters (*P* < 0.001). After 12 and 14 days of storage, the *A*_580_ − *A*_630_ and *I*_SO2_ values were significantly higher in the vitamin‐E‐supplemented lamb meat (0.25, 0.50, or 1.0 g kg^−1^ feed) compared with the meat of the control and rosemary‐supplemented lambs (RE and FRE). For meat discoloration (*A*_580_ − *A*_630_), Renerre and Mazuel^47^ proposed a value of 12.5 as the lower limit for colour acceptability. In the current study, apart from the vitamin‐E‐supplemented lambs that had *A*_580_ − *A*_630_ values higher than 12.5 until 14 days of display, the other treatments only presented acceptable *A*_580_ − *A*_630_ values until day 9 of storage. Similarly, LTL muscle from lambs supplemented with vitamin E presented *I*_SO2_ values higher than 21.0 after 14 days of storage; in contrast, in lambs fed the control or the rosemary‐supplemented diets (RE and FRE), higher *I*_SO2_ values than 21.0 were only maintained for 7 days of storage.

**Table 7:** Effect of dietary supplementation^a^ with *all‐rac*‐*α*‐tocopheryl acetate (SE), rosemary extract (RE), and fat‐embedded rosemary extract (FRE) on meat discoloration (*A*_580_ − *A*_630_ and *I*_SO2_) in raw lamb meat stored in modified‐atmosphere packaging (70% O_2_:30% CO_2_) kept for 1, 7, 9, 12, and 14 days under retail conditions

	Days of display						*P*‐value display
Item	1	7	9	12	14	SEM	
***A***_**580**_ **− *A***_**630**_							
Control	34.66^z^	30.94^z^	19.29^a,y^	8.88^a,x^	7.60^a,x^	2.14	<0.001
SE 250	38.16^z^	38.52^z^	36.84^c,z^	33.19^b,xy^	29.48^b,x^	0.99	<0.001
SE 500	35.98^z^	36.97^z^	34.54^bc,yz^	31.05^b,xy^	28.93^b,x^	1.07	<0.001
SE 1000	37.50^z^	36.96^z^	35.35^bc,yz^	32.94^b,xy^	31.02^b,x^	1.02	<0.001
RE 200	36.84^z^	28.43^z^	17.77^a,y^	9.00^a,x^	7.55^a,x^	2.31	<0.001
RE 400	36.76^z^	28.47^z^	17.60^a,y^	6.80^a,x^	6.09^a,x^	2.05	<0.001
RE 800	36.55^z^	32.41^z^	19.25^a,y^	8.41^a,x^	5.93^a,x^	1.71	<0.001
FRE 200	38.36^z^	29.19^yz^	22.47^a,xy^	13.42^a,wx^	9.74^a,w^	2.12	<0.001
FRE 400	34.42^z^	31.18^yz^	24.81^ab,y^	10.86^a,x^	7.61^a,x^	2.07	<0.001
FRE 800	36.76^z^	31.11^z^	20.62^a,y^	8.58^a,x^	5.61^a,x^	1.66	<0.001
P‐value treatment	0.138	0.124	<0.001	<0.001	<0.001		
***I***_**SO2**_							
Control	19.28^yz^	21.27^z^	20.69^yz^	15.32^abc,xy^	10.62^a,x^	1.38	<0.001
SE 250	18.64^x^	21.54^y^	21.46^y^	21.25^bc,y^	21.08^b,y^	0.33	<0.001
SE 500	16.57^x^	22.38^y^	21.86^y^	21.82^c,y^	20.76^b,y^	0.53	<0.001
SE 1000	17.44^x^	21.80^y^	21.85^y^	21.67^c,y^	21.77^b,y^	0.34	<0.001
RE 200	17.99^yz^	20.94^z^	18.33^yz^	12.51^a,xy^	8.35^a,x^	1.45	<0.001
RE 400	17.76^y^	21.51^y^	18.60^y^	10.91^a,x^	7.20^a,x^	1.21	<0.001
RE 800	17.90^yz^	21.56^z^	18.79^yz^	14.31^ab,xy^	9.04^a,x^	1.18	<0.001
FRE 200	19.07^yz^	21.51^z^	20.12^yz^	15.65^abc,xy^	12.15^a,x^	1.28	<0.001
FRE 400	17.83^xy^	21.03^y^	19.76^y^	16.36^abc,xy^	12.26^a,x^	1.51	<0.001
FRE 800	17.92^yz^	21.36^z^	20.92^z^	14.25^abc,xy^	10.11^a,x^	1.06	<0.001
*P*‐value treatment	0.990	0.808	0.790	<0.001	<0.001		

a SE 250, SE 500, and SE 1000: 250, 500, and 1000 mg *all‐rac*‐*α*‐tocopheryl acetate kg^−1^ feed; RE 200, RE 400, and RE 800: 200, 400, and 800 mg rosemary extract kg^−1^ feed; FRE 200, FRE 400, and FRE 800: 200, 400, and 800 mg fat embedded rosemary extract kg^−1^ feed.

^a,b,c^ Values within a column with different superscript are significantly different (*P* < 0.05). ^w,x,y,z^ Values within a row with different superscript are significantly different (*P* < 0.05). SEM: standard error of the mean.

Lastly, due to the lack of response of both RE sources (RE and FRE) on lipid oxidation, colour stability, myoglobin oxidation, and meat discoloration parameters when compared with the control group, it is not possible to draw any conclusions regarding the efficacy of protection intended by fat embedment.

## CONCLUSIONS

This study demonstrated that feeding lambs a concentrate diet supplemented with *all‐rac*‐*α*‐tocopheryl acetate for 14 days before slaughter resulted in a general improvement in meat oxidative and colour stabilities. This study demonstrates also that, unlike vitamin E, supplementation of lamb diets with an RE (dosage and fat embedment) had no effect on lipid oxidation or meat colour stability of lambs during the 14 days of storage under retail conditions.

## CONFLICT OF INTERESTS

The authors’ contributions are as follows: LNL was the principal investigator and contributed to the study design, data analyses, and interpretation of the findings and wrote the manuscript; JMB contributed to the study design and data collection; JM‐T, LAdH, and WHH contributed to the study design and data interpretation; JAB and MB contributed to the meat analysis and data interpretation. All authors read and approved the final version of the manuscript. WHH, JAB, and MB have no conflict of interests. LNL, JMB, JM‐T, and LAdH are employed by Trouw Nutrition, an animal nutrition company.
